# A Biophysical Model of the Mitochondrial Respiratory System and Oxidative Phosphorylation 

**DOI:** 10.1371/journal.pcbi.0010036

**Published:** 2005-09-09

**Authors:** Daniel A Beard

**Affiliations:** Biotechnology and Bioengineering Center, Department of Physiology, Medical College of Wisconsin, Milwaukee, Wisconsin, United States of America; University of California at San Diego, United States of America

## Abstract

A computational model for the mitochondrial respiratory chain that appropriately balances mass, charge, and free energy transduction is introduced and analyzed based on a previously published set of data measured on isolated cardiac mitochondria. The basic components included in the model are the reactions at complexes I, III, and IV of the electron transport system, ATP synthesis at F_1_F_0_ ATPase, substrate transporters including adenine nucleotide translocase and the phosphate–hydrogen co-transporter, and cation fluxes across the inner membrane including fluxes through the K^+^/H^+^ antiporter and passive H^+^ and K^+^ permeation. Estimation of 16 adjustable parameter values is based on fitting model simulations to nine independent data curves. The identified model is further validated by comparison to additional datasets measured from mitochondria isolated from rat heart and liver and observed at low oxygen concentration. To obtain reasonable fits to the available data, it is necessary to incorporate inorganic-phosphate-dependent activation of the dehydrogenase activity and the electron transport system. Specifically, it is shown that a model incorporating phosphate-dependent activation of complex III is able to reasonably reproduce the observed data. The resulting validated and verified model provides a foundation for building larger and more complex systems models and investigating complex physiological and pathophysiological interactions in cardiac energetics.

## Synopsis

Cells are able to perform tasks that consume energy (such as producing mechanical force in muscle contraction) by using chemical energy delivered in the form of a chemical compound called adenosine triphosphate, or ATP. Two Nobel Prizes were awarded (in 1978 to Peter D. Mitchell and in 1997 to Paul D. Boyer and John E. Walker) for the determination of how ATP is synthesized from the components adenosine diphosphate (ADP) and inorganic phosphate in a subcellular body called the mitochondrion. The operating theory, called the chemiosmotic theory, describes how a driving force called the proton motive force, which arises from the sum of contributions from the electrical potential and the hydrogen ion concentration difference across the mitochondrial inner membrane, is developed by reactions catalyzed by certain enzymes and consumed in generating ATP. Yet, to date, no computer model has successfully described the development and consumption of both the chemical and electrical components of the proton motive force in a thermodynamically balanced simulation. Beard introduces such a model, which is extensively validated based on previously published sets of data obtained on isolated mitochondria. The model is used to test hypotheses about how intracellular respiration is regulated; this model could serve as a foundation for investigating the control of mitochondrial function and for developing larger integrated simulations of cellular metabolism.

## Introduction

As the key cellular organelle responsible for transducing free energy from primary substrates into the ATP potential that drives the majority of energy-consuming processes in a cell, the mitochondrion plays a central role in the majority of eukaryotic intracellular events. Therefore, the development of a quantitative mechanistic understanding of cellular function must rely on a reasonable quantitative description of mitochondrial function. Additionally, development of computational models of physiological systems that span multiple scales, from intracellular biochemistry to whole-organ function, requires a self-consistent integrated description of the biophysical processes observed at the molecular, cellular, tissue, and whole-organ levels of resolution.

Recognizing the need for a computational model of mitochondrial energetics and the need that such a model be available for integration with other physiological systems models, a computational model of the biophysics of the respiratory system and oxidative phosphorylation was developed to meet the following requirements: (1) the model must be consistent with the available experimental data, and (2) the model must be constrained by the relevant physics/biophysics. The utility of the first requirement is self-evident. The second requirement is that models must obey applicable physical laws (e.g., conservation of mass, charge, and energy; the Second Law of Thermodynamics). The alternative to the second requirement is to use empirically derived relationships that are often useful in developing data-driven models based on specific datasets. This approach is not used here because the resulting models often fail when combined together. Physics-based models, on the other hand—i.e., models built on principles including the laws of mechanics and thermodynamics, in which assumptions and approximations are made explicit—operate with a common currency of mass, charge, energy, and momentum [1–3]. Such models naturally integrate across disparate scales.

Previous models of oxidative phosphorylation fail to meet either one or both of the above requirements. For example, the widely used model of Korzeniewski and Zoladz [4–6] invokes an empirical linear relationship between the difference in pH across the mitochondrial inner membrane (matrix pH minus cytosol pH) and the magnitude of inner membrane potential. While the Korzeniewski model has been validated and verified based on a number of studies and is widely applied [7–9], the central empirical relationship between electrostatic potential and pH difference is not expected to apply under all conditions. For example, in the extensive study of isolated cardiac mitochondria published by Bose et al. [10], this relationship is not obeyed. In fact, not only is the linear relationship violated, it is observed in the study of Bose et al. [10] that the matrix pH is nearly constant and can even drop as the magnitude of membrane potential increases. For example, the matrix can become more acidic as the magnitude of the membrane potential increases. This phenomenon cannot be explained by a model that collapses the proton concentration gradient, the membrane potential, and the proton motive force into a single state variable.

The model developed by Magnus–Keizer [11] was integrated by Dudycha and Jafri and colleagues into a detailed model of mitochondrial metabolism, including the reactions of the TCA cycle [12,13]. The Dudycha–Jafri model has been adopted by Cortassa and co-workers and recently extended to account for the production of reactive oxygen species by the respiratory chain [14,15]. The Magnus–Keizer model, developed based on Hill's formalism for biochemical kinetics and free energy transduction [16], is self-consistent and thermodynamically balanced. While it has the advantage over the Korzeniewski model in that the electrostatic potential is treated as a state variable, charge is balanced, and bulk electroneutrality is obeyed in the steady state, the Magnus–Keizer model treats the pH gradient across the inner membrane as a constant. Also, the Magnus–Keizer model addresses certain aspects of mitochondrial ion transport—specifically the transport of calcium across the inner membrane—that are not included at the present stage in the current model. However, details that are unique to the present model—specifically pH buffering by potassium ion exchange [17,18]—are necessary to analyze the extensive experimental dataset considered here.

The goal of the current work is to introduce a quantitative model representing the chemiosmotic theory of the respiratory chain and ATP synthesis that treats the proton gradient and mitochondrial membrane potential as distinct state variables. To be of maximal utility, this biophysically based model of mitochondrial respiration must conserve charge, mass, and energy, and use a treatment of the electron transport system, ATP synthesis, and substrate transporters that does not violate the laws of thermodynamics. The model is developed based on the dataset of Bose et al. [10]. Values of 16 adjustable parameters are estimated based on fitting a relatively large number of experimentally measured data curves (nine data curves). The resulting parameterized model is further validated by comparison to data measured at low oxygen level in isolated mitochondria by Wilson et al. [19] and Gnaiger and Kuznetsov [20,21].

## Results

This section is organized as follows. First, a thermodynamically balanced biophysical model of mitochondrial oxidative phosphorylation is developed, along with identification of model parameters based on the measured dependence of NADH and cytochrome c redox states, oxygen consumption, and matrix pH on levels of buffer phosphate. It is shown that the developed model cannot match data on the inner membrane electrostatic potential without incorporating phosphate-dependent control of oxidative phosphorylation. In the next section, the isolated mitochondrial model is modified to include phosphate-dependent activation of complex III and is fit to the data on inner membrane potential. In the final section, the behavior of the model at low oxygen level is shown to compare favorably to data reported by Gnaiger and Kuznetsov [20,21] and Wilson et al. [19].

### Mitochondrial Model without Phosphate Control

The basic components of the mitochondrial model, which include the reactions at complexes I, III, and IV of the respiratory chain and ATP synthesis at F_1_F_0_ ATPase, are illustrated in Figure 1A. Substrate transporters, including adenine nucleotide translocase (ANT) and the phosphate–hydrogen co-transporter (PiHt) are illustrated in Figure 1B. Cation fluxes across the inner membrane, illustrated in Figure 1C, include fluxes through the K^+^/H^+^ antiporter and passive H^+^ and K^+^ permeation. The model includes two mitochondrial compartments (matrix and intermembrane [IM] space). In the following description of the model equations, subscripts “x”, “i”, and “e” denote concentrations of reactants in the matrix, IM space, and external space, respectively; the concentration variables [fATP]_i_ and [fATP]_x_, and [fADP]_i_ and [fADP]_x_, denote the magnesium-unbound components of ATP in the IM space and the matrix, and unbound ADP concentrations in the IM space and matrix, respectively; variables [mATP]_i_ and [mATP]_x_, and [mADP]_i_ and [mADP]_x_, denote the magnesium-bound components of ATP in the IM space and the matrix, and magnesium-bound ADP concentrations in the IM space and matrix, respectively. The external space corresponds to the extra-mitochondrial buffer used in the experiments simulated below.

**Figure 1:**
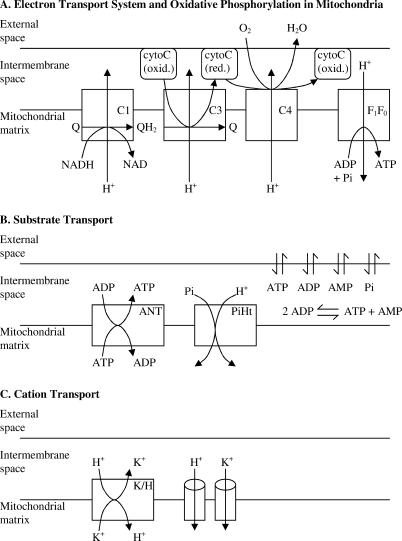
Illustration of the Components Included in the Model of Mitochondrial Oxidative Phosphorylation (A) The major components of the electron transport system, which transfers reducing potential from NADH to oxygen, and the F_1_F_0_ ATPase, which transduces energy from proton motive force to ATP, are illustrated. Complexes I, III, and IV are labeled C1, C3, and C4, respectively. (B) The substrate transport process included in the model is shown, including the ANT and PiHt on the inner membrane, and passive permeation of ATP, ADP, AMP, and phosphate across the outer membrane. The AK reaction in the IM space is shown. (C) Transporters for hydrogen and potassium ions on the inner membrane, including K^+^/H^+^ antiporter and passive proton and potassium fluxes, are included. It is assumed that these cations rapidly equilibrate across the outer membrane.

#### Note on units.

All concentrations in the following sections are expressed in molar units—specifically moles per liter of compartment volume. Fluxes are expressed in units of mass per unit time per unit mitochondrial volume. To avoid confusion, the units on flux variables are written as “mol s^−1^ (l mito volume)^−1^” and not simplified to “M s^−1^”, which would be ambiguous when referring to fluxes for membrane transporters.

#### Dehydrogenase flux.

In this model the TCA cycle and other NADH-producing reactions are not explicitly modeled. Instead, a phenomenological driving force is used to simulate the phosphate-dependent rate of reduction of NAD^+^ to NADH via the reaction NAD^+^ 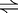 NADH + H^+^ in the mitochondrial matrix. The following expression is used to model the dehydrogenase flux:


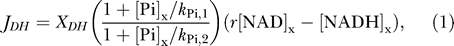


where [NADH]_x_ and [NAD]_x_ denote the concentrations of NADH and NAD in the mitochondrial matrix; [Pi]_x_ denotes the matrix inorganic phosphate concentration, and *X_DH_, r, k*_Pi,1_, and *k*_Pi,2_ are empirical parameters. Thus, the dehydrogenase flux drives the [NADH]_x_/[NAD]_x_ ratio toward *r,* with a reaction rate dependent on the concentration of phosphate, which serves as a substrate for mitochondrial dehydrogenase enzymes.

#### Electron transport system fluxes.

The overall reaction for electron transfer from NADH to ubiquinol at complex I is expressed as


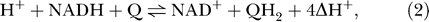


where the notation ΔH^+^ is used to indicate hydrogen ion transferred from the matrix to the cytosol, against the electrochemical gradient. The direction of positive flux for the reaction of equation 2 and for all reactions introduced below is left-to-right.

The flux through complex I is modeled using the following expression:


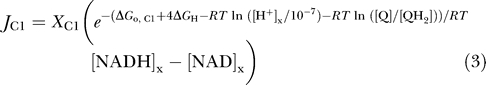


where *X*_C1_ is an adjustable parameter, [Q] and [QH_2_] denote oxidized and reduced ubiquinol, *RT* is the gas constant multiplied by the absolute temperature; ΔG_o,C1_ = −69.37 kJ mol^−1^ is the standard free energy for the reaction H^+^ + NADH + Q 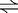 NAD^+^ + QH_2_ at pH = 7; and Δ*G*_H_ is the proton motive energy, or the free energy change associated with pumping a proton from the matrix side to the cytosol side of the mitochondrial inner membrane. The factor of four in the exponent of equation 3 arises from the four protons pumped from the matrix space to the IM space for each pair of electrons transferred from NADH to ubiquinol. The proton motive energy is computed as follows:


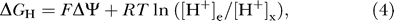


where *F* is Faraday's constant, ΔΨ is the membrane potential measured as the outer potential minus the inner potential, and [H^+^]_e_/[H^+^]_x_ is the ratio of external hydrogen ion concentration to matrix concentration. Equation 3 represents a minimal thermodynamically balanced one-parameter model for the flux through the first step in respiratory system—i.e., the flux described by equations 3 and 4 drives concentrations towards thermodynamic equilibrium. Specifically, the flux is driven towards a thermochemical equilibrium defined by the effective equilibrium expression


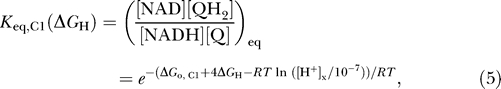


which is an explicit function of the proton gradient and electrostatic gradient across the inner membrane.

For complex III, it is assumed that four protons are pumped for each pair of electrons transferred from ubiquinol to cytochrome c [22,23]:


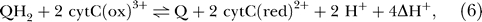


where cytC(ox)^3+^ and cytC(red)^2+^ denote the oxidized and reduced forms of cytochrome c, respectively. As indicated in Figure 1, cytochrome c is assumed to be present in the IM space. Although four protons are pumped across the inner membrane for each unit flux through this reaction, the total number of charges transferred is two, owing to the redox transfer from ubiquinol to cytochrome c, which generates two matrix hydrogen ions for each turnover of the reaction. The flux through complex III takes a form similar to equation 3:


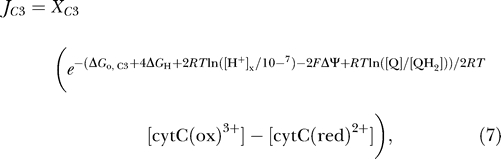


where *X*_C3_ is an adjustable parameter, ΔG_o,C3_ = −32.53 kJ mol^−1^. Below, the expression for complex III flux is modified to test the hypothesis that phosphate modulates complex III activity. It will be shown that, while much of the available experimental data can be explained by the model developed in this section, which does not consider phosphate-dependent control of the respiratory chain enzymes, the observed data are better fit by a model that incorporates phosphate-dependent control of complex III activity.

The overall reaction of complex IV involves the transfer of two protons across the membrane and a total of four charges [24,25]:


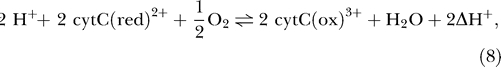


with a flux computed as


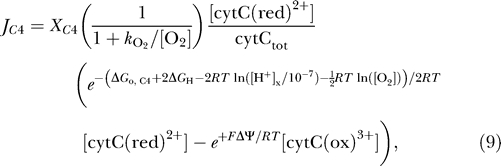


where *X*_C4_ is an adjustable parameter, ΔG_o,C4_ = −122.94 kJ mol^−1^, and cytC_tot_ = [cytC(ox)^3+^] + [cytC(red)^2+^].

The complex IV flux of equation 9 is expressed in a form similar to those of complexes I and III, with a few key differences. While, as for complexes I and III, the flux is formulated to drive the system toward thermodynamic equilibrium, additional multiplicative factors have been included in order to successfully reproduce the observed data. The factor 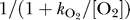 is included in equation 9 to account for the observed dependence of the rates of oxygen consumption and ATP generation on oxygen concentration [6,19–21,26,27]. It will be shown that the factor [cytC(red)^2+^]/cytC_tot_ is found to provide better fits to the observed data than are possible without it.

#### ATP synthesis.

ADP is phosphorylated to ATP in the matrix via the F_1_F_0_-ATPase reaction:


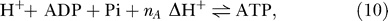


where *n_A_* ≈ 3 is the number of protons transported each time this reaction turns over. Since ATP synthesis requires magnesium as a cofactor, the flux through this complex is modeled using the thermodynamically balanced expression


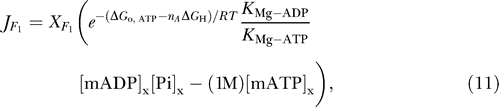


where *X*_F1_ is an adjustable parameter, ΔG_o,ATP_ = −36.03 kJ mol^−1^ and *K*_Mg-ATP_ and *K*_Mg-ADP_ >are the equilibrium dissociation constants for ATP and ADP binding with Mg^2+^. The factor 1 M multiplying [mATP]_x_ is used so that the term in parenthesis is balanced in terms of units.

#### Magnesium binding.

Binding between magnesium ion and ATP and ADP is driven via the following fluxes:


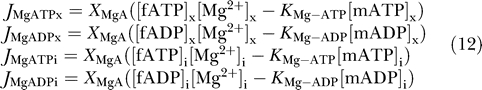


fATP and fADP denote the concentrations of ATP and ADP that are not bound to magnesium ion in the matrix and IM space; mATP and mADP denote the magnesium-bound species. The parameter *X*_MgA_ is the forward binding rate constant for these reactions; the effective unbinding constant is computed to satisfy the equilibrium dissociation relations for ATP and ADP binding with Mg^2+^.

#### Substrate transport.

Permeation of ATP, ADP, AMP, and inorganic phosphate between the external buffer and the IM space is governed by the following fluxes:


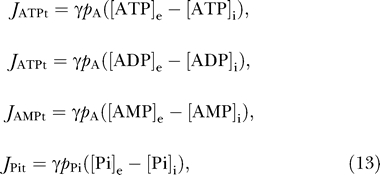


where the subscripts “e” and “i” denote external buffer and IM space, respectively. The buffer concentrations are set as constants in this study. The permeabilities of the outer membrane to adenine nucleotides and to inorganic phosphate are given by *p*_A_ and *p*_Pi_, respectively; γ denotes the ratio of mitochondrial outer membrane area to total cardiomyocyte cell volume.

ANT flux involves the displacement of one negative charge from the matrix to the IM space, and is therefore coupled to the electrostatic membrane potential. The following empirical expression [4,6] is used to model the ANT flux:


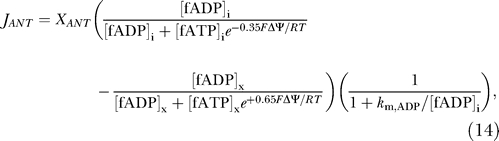


where the ANT is assumed to operate on magnesium-unbound ATP and ADP in the two compartments.

Transport of inorganic phosphate between the matrix and IM space is coupled to the hydrogen ion gradient [28]. It is assumed that H^+^ and 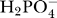 are transported by a co-transport process, with H^+^ and 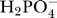 moving together across the membrane in a 1:1 ratio in a net electroneutral exchange. Hydrogen binding to inorganic phosphate via the reaction


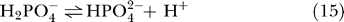


is assumed to be in equilibrium on either side of the membrane with 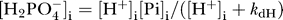 and 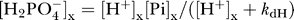 , where *k*_dH_ is the dissociation constant for the reaction. In these expressions Pi represents the sum of species 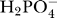 and 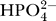 . The phosphate-hydrogen co-transporter flux is modeled as reversible Michaelis–Menten flux:


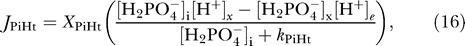


where *X*_PiHt_ is an adjustable parameter, *k*_PiHt_ is the Michaelis–Menten constant for 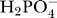 on the outside of the membrane.

#### Adenylate kinase reaction.

High-energy phosphates are transferred between ATP, ADP, and AMP in the IM space via the Adenylate kinase (AK) reaction:


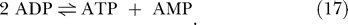


The AK flux in the IM space is computed as follows:


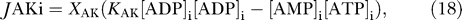


where *K*_AK_ = 0.4331 is the equilibrium constant for the reaction of equation 17, and *X*_AK_ is the AK enzyme activity.

#### Cation transport.

The present work assumes that calcium and sodium concentrations and fluxes have only secondary effects on membrane potential compared to the primary effects of currents associated with the respiratory chain, the ANT current, and the proton leak. Therefore, fluxes of sodium and calcium are not considered at this stage. Since K^+^ is required to buffer the matrix pH [17] and Mg^2+^ is required for ATP synthesis and the ANT flux, these ions are considered in the model. Expressions for K^+^ and Mg^2+^ channel and transporter fluxes are developed below.

The expression for the leak of H^+^ across the inner membrane is obtained by solving the one-dimensional Nernst–Planck equation, the differential equation for diffusion and drift of a charged species across a permeable membrane. The resulting flux is calculated from the Nernst–Goldman equation [29,30]:


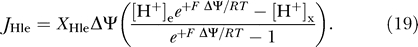


Passive flux of potassium into the matrix is modeled using a similar expression:


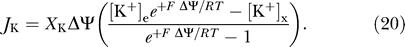


While significant evidence exits for passive flux of potassium through various channels into the matrix [17,31], it is unclear exactly what transporters are present to prevent potassium concentrations from approaching thermochemical equilibrium across the inner membrane. It is assumed that the outflow of potassium ions from the matrix is coupled to the proton gradient, and outflow is modeled using a simple reversible antiporter with flux given by mass-action kinetics:


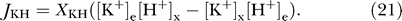


The above expressions for K^+^ and H^+^ transport assume that these ions rapidly equilibrate across the outer membrane. Therefore, the IM space concentrations are assumed to be equal to the external space concentrations.

#### Governing equations.

The flux expressions are used to construct a kinetic model for the system; the overall system is governed by the following set of 17 differential equations:


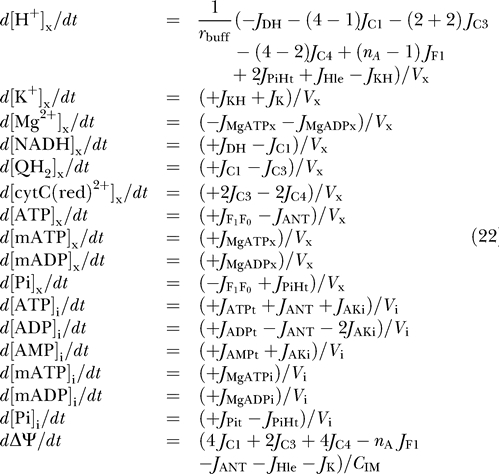


where *V*_x_ and *V*_i_ are the matrix and IM space water volumes, and *r*_buff_ is the buffering capacity of the matrix space, which is set to *r*_buff_^−1^ = (100 M^−1^) · [H^+^]_x_ [32].

The stoichiometric coefficients multiplying the complex I, III, and IV, and F_1_F_0_-ATPase fluxes include two terms, one term representing the number of protons transported across the inner membrane for a given reaction, and one term representing the number of protons consumed by the associated biochemical reaction. For example, the complex III reaction pumps four H^+^ out of the matrix and consumes one matrix H^+^ for every turnover of the reference biochemical reaction H^+^ NADH + Q 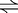 NAD^+^ + QH_2_, resulting in a total of three matrix H^+^ consumed. Thus, the net stoichiometric coefficients multiplying *J*_C1_, *J*_C3_, *J*_C4_, and *J*_F1_ are −(4 − 1), −(2 + 2), −(4 − 2), and +(*n_A_* − 1).

The membrane potential kinetics depends on the effective membrane capacitance, *C*_IM_, which is estimated below.

In addition to the 17 state variables treated in equation 22, the concentrations of oxidized matrix NAD, Q, and cytochrome c, and the matrix ADP concentration, are computed as follows:


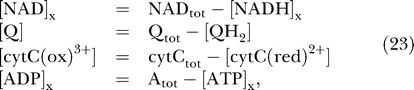


where NAD_tot_, Q_tot_, cytC_tot_, and A_tot_, are the total concentrations of NAD(H), ubiquinol, cytochrome c, and adenine nucleotide in the matrix, respectively.

#### Parameter values.

The values of the parameters used in this section are listed in Table 1. The units on activities are expressed as mass flux per unit time per unit total mitochondrial volume, specifically mol s^−1^ (l mito volume)^−1^. The parameters have been categorized into three classes, denoted classes A, B, and C. The meaning of these categories is as follows.

**Table 1:**
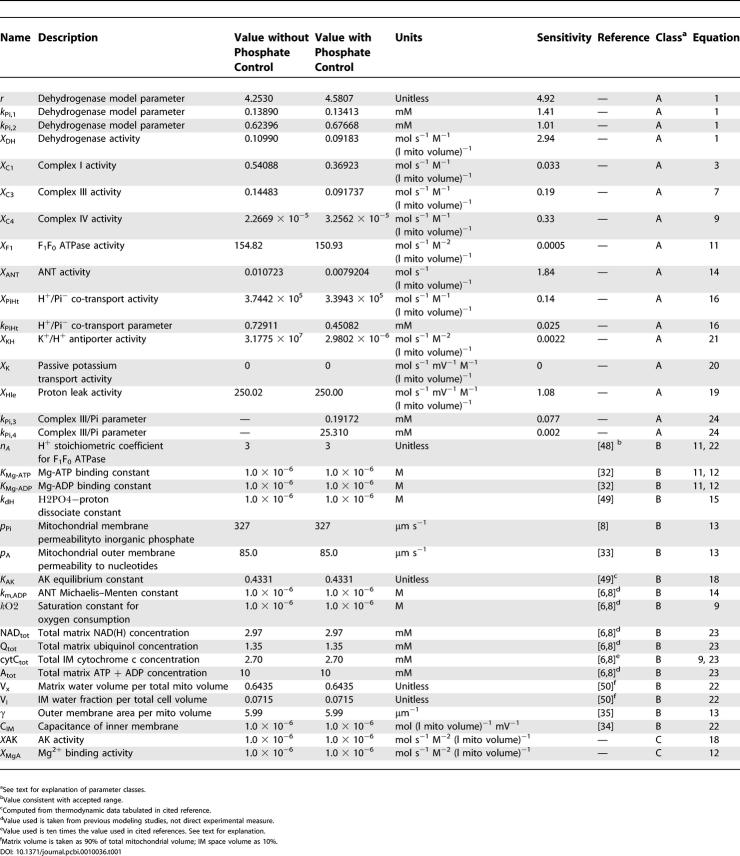
Mitochondrial Model Parameter Values

Class A refers to free parameters with values determined by fitting model simulations to the data published by Bose et al. [10]. In total, there are 14 adjustable parameters, which are estimated by fitting to seven data curves (described below.) Of the 14 adjustable parameters, four correspond to the phenomenological model of dehydrogenase flux, while the remaining ten are associated with the biophysical model of oxidative phosphorylation and electron transport system in cardiac mitochondria.

Class B refers to 17 parameters for which values are established in the literature. These parameter values were fixed and not treated as adjustable. The values used for NAD_tot_, A_tot_, Q_tot_, and cytC_tot_ are obtained from the previous models of Vendelin et al. [8] and Korzeniewski and Zoladz [4,6]. The current model assumes cytochrome c to be distributed within the IM space, in contrast to previous models, in which cytochrome c is in the matrix. To keep the total mass of cytochrome c consistent, cytC_tot_ is set to 2.7 mM, a value that is ten times greater than that used in the Vendelin and Korzeniewski models, since the IM volume is assumed to be 1/10 of the mitochondrial water volume. The value for the outer membrane permeability to adenine nucleotides is estimated from Lee et al. [33]. Assuming the mitochondrial inner membrane has a capacitance of 1 μF per square centimeter of surface area [34], the inner membrane capacitance is calculated to be 6.75 × 10^−6^ mol (l mito volume)^−1^ mV^−1^ for an inner membrane area of 60 μm^2^. It is observed that the steady-state model behavior presented below is not sensitive to the assumed value of mitochondrial membrane capacitance. The steady-state membrane potential is determined by bulk electroneutrality, which imposes the constraint that the sum of the various currents across the membrane is zero. Thus, 4*J*_C1_ + 2*J*_C3_ + 4*J*_C4_ − *n_A_J*_F1_ −*J*_ANT_ − *J*_Hle_ − *J*_K_ − 2*J*_Mg_ = 0, where each of these fluxes depends on the membrane potential. The ratio of mitochondrial surface area to volume γ is estimated from morphological data [35] to be 5.99 μm^−1^. Given this value of γ and the assumed values of outer membrane permeability, the gradients obtained for ATP and ADP across the outer membrane at maximal respiration rate are 20 μM. In this range, the resistance to passive transport between the inner membrane space and buffer is not great enough to be significant and the model behavior is not sensitive to the assumed value of γ.

Class C refers to two parameters that are set to extreme values such that the simulated model behavior is not sensitive to the specific value chosen. The AK and magnesium-binding activities are set to values high enough that the corresponding reactions maintain equilibrium.

Table 2 lists the values for the standard free energies for the reactions of the respiratory chain at pH = 7.

**Table 2:**
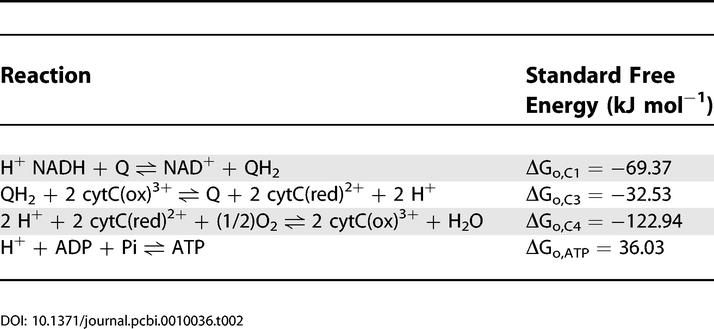
Standard Free Energies of Respiratory Chain Reactions

#### Simulation of isolated mitochondria.

The extensive dataset published by Bose et al. [10] is used to parameterize the mitochondrial model. The external K^+^, H^+^, ATP, ADP, AMP, and inorganic potassium concentrations were set as constants according to the buffer concentrations imposed in the experiment in order to compare model simulations to the experimental data. Specifically, [H^+^]_e_ = 10^−7.1^, [ATP]_e_ = 0, and [AMP]_e_ = 0; [ADP]_e_ was set at either 0 or 1.3 mM, as described below, and [Pi]_e_ was varied from 0 to 10 mM. The total magnesium concentration in the buffer was fixed at 5.0 mM; buffer potassium concentration was fixed at 150 mM. The simulations described in this section were computed with the oxygen partial pressure in the matrix set to 20 mm Hg, or [O_2_] = 2.6 × 10^−5^ M.

The black curves plotted in Figure 2 illustrate comparisons between model-simulated and experimentally measured values for the dataset used to estimate the 14 adjustable parameters listed in Table 1. Parameters values were adjusted to obtain the best fit (least squares error) between model simulations and experimental measures for steady-state values of NADH concentration, rate of oxygen consumption, cytochrome c redox state, and matrix pH, as shown in the figure. Optimal parameter values were found using a global Monte-Carlo-based simulated annealing algorithm that searched for the optimal set of parameter values to simultaneously fit several data curves. In total, seven independent curves were used to estimate the 14 parameter values. It was found that best-fit model solutions are obtained by setting the passive potassium and magnesium fluxes to zero. Shown in Figure 2A are model simulations of steady-state NADH (normalized to NAD_tot_) as a function of external inorganic phosphate, [Pi]_e_. The two curves correspond to two different values of external ADP concentration, 0 and 1.3 mM, as indicated in the figure. Also shown are data from Bose et al. [10], collected from isolated mitochondria suspensions, with buffer ADP concentrations of 0 mM (circles) and 1.3 mM (triangles). Figure 2B illustrates the experimentally measured and model-simulated values of the rate of oxygen consumption (MV_O2_) for the same conditions as described for the NADH curves in Figures 2A. When substrate concentration (either ADP or inorganic phosphate) goes to zero, the nonzero MV_O2_ corresponds to the basal oxygen consumption necessary to maintain the proton motive energy with a finite proton leak across the inner membrane. Figure 2C illustrates the model-simulated and experimentally measured values of cytochrome c redox state; Figure 2D illustrates the model-simulated and experimentally measured values of matrix pH. The matrix pH is buffered at a nearly constant value via H^+^/K^+^ exchange.

**Figure 2:**
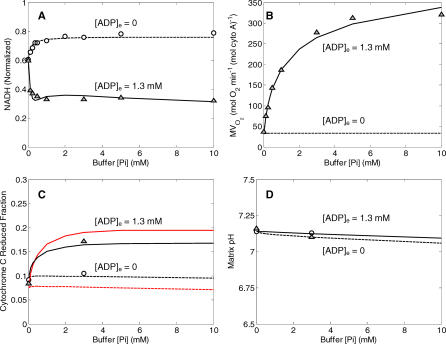
Comparison of Model Simulations to Experimental Data on NADH, MV_O2_, Cytochrome C Redox, and Matrix pH for Model without Phosphate Control (A) Results for normalized matrix NADH as a function of buffer inorganic phosphate concentration are shown for the two experimental cases of resting mitochondria ([ADP]_e_ = 0, state 4) and active state mitochondria ([ADP]_e_ = 1.3 mM, state 3). (B) Results for MV_O2_ (rate of oxygen consumption) are shown for the same experimental cases as in (A). Experimental data are not available for the resting state, in which a minimal flux through the electron transport system is maintained to compensate for cation flux across the inner membrane. (C) Results for cytochrome C reduced fraction are shown for the experimental cases as in (A). The black curves correspond to the model equations developed in the text. The red curves correspond to the best-fit model simulations obtained with equation 9 modified to not include the factor [cytC(red)^2+^]/cytC_tot_ multiplying the expression for *J*_C4_. (D) Matrix pH (model-simulated and experimentally measured) is plotted as a function of buffer phosphate for the experimental cases as in (A). All computed results in this figure correspond to steady-state simulations of model described under “Mitochondrial Model without Phosphate Control.” Model simulations for [ADP]_e_ = 1.3 mM, and [ADP]_e_ = 0 mM are plotted as solid lines and dashed lines, respectively. Experimental data (circles and triangles) are obtained from [10].

Also shown as red curves plotted in Figure 2C is the best fit to the cytochrome redox state data obtained without the factor cytC(red)^2+^]/cytC_tot_ multiplying the *J*_C4_ flux expression of equation 9. It is observed that the model's fits to the cytochrome c redox data are improved by incorporating this multiplicative factor in the expression for complex IV flux. Thus the best-fit model solution assumes the flux through complex IV depends on [cytC(red)^2+^]^2^, which may be explained by the fact that two reduced cytochrome c molecules are require to donate a single electron pair one oxygen atom, generating H_2_O.

In contrast to the results illustrated in Figure 2, the model described in this section is unable to reproduce data on mitochondrial membrane potential. In Figure 3 are plotted the model-simulated and experimentally measured values of ΔΨ, as a function of [Pi]_e_ for the cases of [ADP]_e_ = 0 mM and [ADP]_e_ = 1.3 mM.

**Figure 3:**
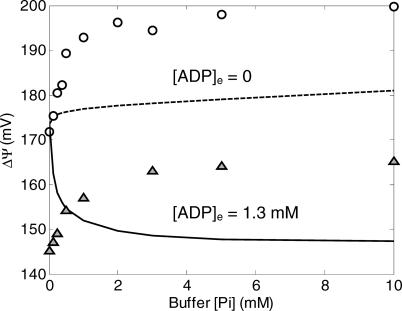
Comparison of Model Simulations to Experimental Data on Membrane Potential for Model without Phosphate Control The model without phosphate control is not able to fit the experimental data on mitochondrial membrane potential. Computed results in this figure correspond to steady-state simulations of the model described under “Mitochondrial Model without Phosphate Control.” Model simulations for [ADP]_e_ = 1.3 mM, and [ADP]_e_ = 0 mM are plotted as solid lines and dashed lines, respectively. Experimental data (circles and triangles) are obtained from [10].

In the inactive state ([ADP]_e_ = 0), as the external (buffer) phosphate concentration is increased, the dehydrogenase flux governed by equation 1 increases, providing an increased thermodynamic driving force for electron transport and a corresponding increase in the magnitude of the membrane potential. However, the model-simulated rate of increase in ΔΨ with [Pi]_e_ is much smaller than that observed experimentally. When the active state is simulated ([ADP]_e_ = 1.3 mM), the model fit is even worse than for the inactive state. The addition of ADP to the external buffer, representing a sink for the free energy stored in the redox state in the matrix and the membrane potential, results in a drop in both redox and membrane potential. The simulated magnitude of the potential difference decreases with increasing [Pi]_e_, the opposite of the trend that is observed experimentally. With 14 adjustable parameters, it is not possible to reproduce the experimentally observed behavior of ΔΨ while maintaining reasonable fits to the curves plotted in Figure 2.

### Mitochondrial Model with Phosphate Control

Reasonable fits to the observed ΔΨ require that phosphate-dependent control be incorporated into the model, as proposed by Bose et al. [10]. It is found that by including phosphate-dependent control of complex III it is possible to obtain improved fits to the data on membrane potential. The flux expression of equation 7 is modified as follows:


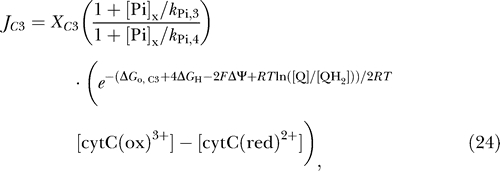


where two new parameters, *k*_Pi,3_ and *k*_Pi,4_, have been introduced. Thus, the total number of adjustable parameters in the model is increased from 14 in the previous section to 16. By varying model parameters, including these additional parameters, we are able to obtain fits to the nine data curves as illustrated in Figures 4 and 5. Thus, the large number of parameters is offset by a relatively large number of data curves to fit for estimation of parameter values. As for the model described in the previous section, it is found that best-fit model solutions are obtained by setting the passive potassium flux to zero. Therefore, in the model parameterization presented in Table 1 the activities of the corresponding channels are set to zero, effectively reducing the number of adjustable parameters. Sensitivity analysis reveals that the activities of the channels cannot be determined based on the present dataset. The activities of potassium channels are known to be modulated by ischemic and anesthetic preconditioning [31,36,37] and will be explored in future work.

**Figure 4:**
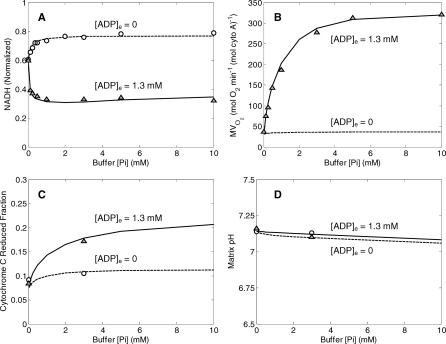
Comparison of Model Simulations to Experimental Data on NADH, MV_O2_, Cytochrome C Redox, and Matrix pH for Model with Phosphate Control (A) Results for normalized matrix NADH as a function of buffer inorganic phosphate concentration are shown for the two experimental cases of resting mitochondria ([ADP]_e_ = 0, state 4) and active state mitochondria ([ADP]_e_ = 1.3 mM, state 3). (B) Results for MV_O2_ (rate of oxygen consumption) are shown for the same experimental cases as in (A). (C) Results for cytochrome C reduced fraction are shown for the experimental cases as in (A). (D) Matrix pH (model-simulated and experimentally measured) is plotted as a function of buffer phosphate for the experimental cases as in (A). All computed results in this figure correspond to steady-state simulations of the model described under “Mitochondrial Model with Phosphate Control.” Model simulations for [ADP]_e_ = 1.3 mM and [ADP]_e_ = 0 mM are plotted as solid lines and dashed lines, respectively; experimental data are the same as plotted in Figure 2.

**Figure 5:**
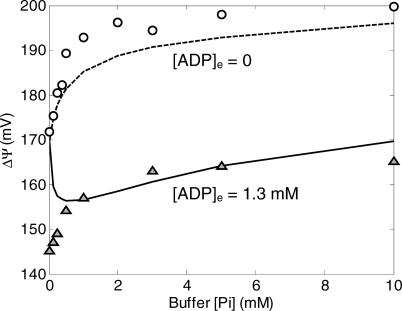
Comparison of Model Simulations to Experimental Data on Membrane Potential for Model with Phosphate Control The model with phosphate control compares much more favorably to the experimental measurements than the model without phosphate control (see Figure 3). Computed results in this figure correspond to steady-state simulations of the model described under “Mitochondrial Model with Phosphate Control.” Model simulations for [ADP]_e_ = 1.3 mM and [ADP]_e_ = 0 mM are plotted as solid lines and dashed lines, respectively; experimental data are the same as plotted in Figure 3.

The fits obtained using equation 24 to model the complex III flux are plotted in Figures 4 and 5. Note that the model simulation of NADH, MV_O2_, cytochrome c state, and matrix pH plotted in Figure 4 produces values similar to those obtained using the previous model (see Figure 2). Of particular note is the behavior of the NADH redox state and MV_O2_ curves, plotted in Figure 4A and 4B. In these cases the mean squared error between observed data and the model fits is slightly lower than that obtained using the model with no phosphate control (see Figure 2A and 2B).

The major difference between the model of this section and that of the previous section is seen in the simulated membrane potential values, plotted in Figure 5. With the phosphate-modulated control described by equation 24, the model remains unable to reproduce the observed data on membrane potential as phosphate concentration goes to zero. Yet while the model's fits to the observed data remain imperfect, the agreement between simulation and experimental data is significantly improved by incorporating the expression of equation 24 to model the complex III flux. Possible mechanisms explaining differences between model simulations and the data observed in the limit [Pi]_e_ → 0 at [ADP]_e_ = 1.3 mM are outlined in the Discussion.

The values of the parameters used in generating Figures 4 and 5 are listed in Table 1. The sensitivities of the estimated values of model parameters are considered in the Discussion.

### Behavior of Model at Low Oxygen Concentration

A key to understanding cellular energetics during hypoxia and ischemia is a mechanistic model of mitochondrial function at low oxygen concentration. In this section the behavior of the model is compared to measurements of oxygen consumption and cytochrome c reduction in isolated mitochondria as functions of the oxygen concentration of the medium.

The behavior of the model at low oxygen concentration is illustrated in Figure 6. Plotted are the rate of mitochondrial oxygen consumption and the predicted reduced fraction of cytochrome c as functions of the oxygen content of the medium.

**Figure 6:**
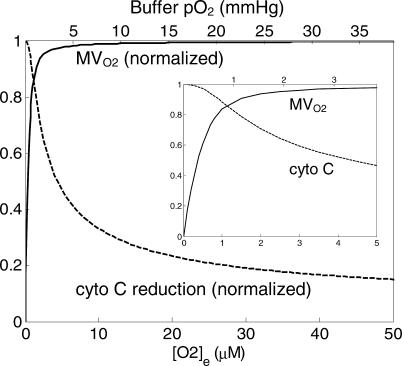
Behavior of Model at Low Oxygen Concentration Predicted rate of oxygen consumption (MV_O2_) normalized to maximal rate of oxygen consumption and fraction of cytochome c reduced are plotted against oxygen concentration, which is expressed in micromoles (lower axis) and oxygen partial pressure (upper axis). The oxygen consumption curve was computed for state-3 respiration, corresponding to experimental conditions reported in [18] and [19]. The cytochrome c curve corresponds to state-4 experimental conditions reported in [17]. Inset shows predicted curves for oxygen concentrations from 0 to 5 μM.

The oxygen consumption curve was computed for active state-3 respiration, with [ADP]_e_ and [ATP]_e_ set to 1.0 mM and 0 mM, respectively. This curve corresponds to the curves reported in Figure 7A of [20] and Figure 2A of [21]. The model-predicted *P*_50_ for half-maximal oxygen consumption is 0.373 μM (or an oxygen partial pressure of 0.287 mm Hg), close to the reported value of 0.35 ± 0.07 μM [20,21]. The curve for the predicted reduced fraction of cytochrome c (dashed line in Figure 6) was computed for state-3 respiration ([ADP]_e_ = 0.5 mM; [ATP]_e_ = 0.87 mM), corresponding to the measurements of Wilson et al. [19]. The predicted curve can be compared to Figures 4B and 5A of [19]. Wilson et al. found that in mitochondria isolated from rat liver, cytochrome c is approximately 15%–18% reduced for oxygen concentration in the range of 40–50 μM. The current model predicts a slightly lower value of 14% reduced at [O_2_]_e_ = 50 μM.

Thus, beyond the dataset used for parameterization, the model was further validated by comparison to additional datasets measured from mitochondria isolated from rat heart [20,21] and liver [19] and observed at low oxygen concentration. The model agrees quantitatively with the measured dependence of oxygen consumption rate on oxygen concentration in state-3 respiration. Although the observed cytochrome c redox state is slightly (1%–4%) more reduced in measurements reported for isolated rat liver mitochondrial at low oxygen concentrations compared with the model predictions, the predicted behavior of cytochrome c reduction relative to oxygen concentration is qualitatively similar to the corresponding experimental observations. Allowing for differences in the behaviors of hepatic and cardiac mitochondria, an exact quantitative agreement may not be expected.

## Discussion

### Model Development and Parameterization

The main contribution of the current study is the introduction of a self-consistent thermodynamically balanced model of oxidative phosphorylation and the electron transport system in mitochondria. A biophysical model incorporating all of the components illustrated in Figure 1 required the development of a system of 17 differential equations and the introduction of 16 adjustable parameters. To identify such a large number of parameters, it was necessary to make use of a large number of independent measurements made on mitochondria isolated from rat cardiac tissue [10]. This previously published dataset consists of measures of NAD(H) redox state, cytochrome c redox state, rate of oxygen consumption, mitochondrial membrane potential, and matrix pH, for a range of buffer conditions, including a range of concentrations of inorganic phosphate and for resting and active state mitochondria ([ADP]_e_ = 0 and 1.3 mM, respectively). In total, 16 parameters were estimated by simultaneously fitted model-simulated steady states to nine independent data curves (see Figures 4 and 5), providing quantitative estimates of the model parameters.

While an exhaustive statistical analysis of the 16-dimensional parameter space is not computationally feasible, it is possible to compute the sensitivities of the mean squared error between the model solutions to the estimated parameter values. Parameter sensitivity can be estimated from the diagonal entries of the Hessian of the error function, 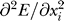 , where *E* is the mean squared difference between model simulations and experimental data and *x_i_* represents *i*th parameter. However, the partial derivatives of *E* with respect to parameter values represent local measures that do not necessarily reflect how the error changes with finite changes in the parameter values. To estimate the sensitivity to finite changes in parameter values, the sensitivity to each parameter was computed as the relative change in mean squared error due to a 10% change in a given parameter value:


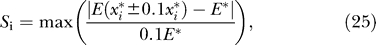


where E* is the minimum mean squared difference between model simulations and experimental data, and 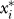 is the optimal value of the *i*th parameter. The term 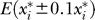 is the error computed while setting parameter *x_i_* to 10% above and below its optimal value. The relative sensitivities to the adjustable parameters are listed in Table 1. These sensitivity values represent a measure of the degree to which the curves plotted in Figures 4 and 5 are sensitive to the value of the individual parameters. A high sensitivity value indicates that changing a given parameter results in significant changes to the simulated curves used to identify the set of adjustable parameter values. Note that six of the adjustable parameters show relative sensitivity of less than 5%, indicating that model solutions are not particularly sensitive to these parameters in the neighborhood of the reported values and that these parameters are not well estimated by the present analysis. In particular, in comparison to the dataset presented here, the model is relatively insensitive to the values of the activities of the potassium transporters and the F_1_F_0_ ATPase. The best model fits were obtained with the potassium channel activities set effectively to zero. Therefore, the relative sensitivity to this parameter is reported to be zero as well. The ATP synthesis activity *X*_F1_ is determined to be large enough that the reaction is effectively maintained in equilibrium. To estimate the parameters that are poorly identified, it will be necessary to obtain appropriate data in the future. In fact, because the model is parameterized based solely on steady-state data, it may not accurately match time-dependent behavior of mitochondrial oxidative phosphorylation. Thus, it is expected that kinetic data, in particular, will be of great value in refining the parameter estimates, refining the model, and generally improving the ability of the model to predict observed behavior.

Yet while the model represents only one component of mitochondrial energy metabolism that one may build on and refine, it does represent the most complete model of the respiratory chain that is available to date. The model includes the major components of oxidative phosphorylation and the electron transport system and appropriately balances mass, charge, and free energy. By integrating the components illustrated in Figure 1 into a self-contained model, the observations of Bose et al. [10] have been explained based on a model that incorporates phosphate control at the dehydrogenase flux (perhaps via mass action) and phosphate-dependent activation of complex III. Of course, the model does not reproduce the experimental data with perfect fidelity. The major shortcomings of the current model analysis are the inability to reproduce the observed data in state-3 mitochondria as buffer phosphate concentration approaches zero and the inability to sensitively identify parameters for membrane ion transporters. As discussed below, the observations at [Pi]_e_ → 0 may be influenced by an experimental artifact; detailed identification of the inner membrane ion transporters will require the design of further experiments sensitive to the kinetic behavior of these channels.

### Phosphate-Dependent Control of the Respiratory Chain

To obtain the model solutions illustrated in Figures 4, 5, and 6, an expression for complex III flux was developed based on the hypothesis that inorganic phosphate level modulates the activity of the complex III. While no data directly measuring the activity of complex III in intact mitochondria as a function of phosphate concentration are available, the hypothesis is supported by the fact that the model's fits to the observed data are significantly improved when phosphate-dependent control is included compared to the case when it is not. In work not detailed, a number of similar control expressions were tested using phosphate and other species (ATP, ADP, Mg^2+^) as putative controllers of complexes I, III, and IV and of F_1_F_0_ ATPase and the ANT system. It was found that no other choice of controlled enzyme and controller species could provide fits to the data of Figure 5 as reasonable as that of the hypothetical control of complex III by inorganic phosphate. Thus, 20 independent hypotheses (each formulated as the activity of one of five enzymes dependent on one of four species) were quantitatively tested and 19 were excluded as not able to reproduce the observed data. Yet, by increasing the complexity of the model, in particular by hypothesizing phosphate-dependent control of a more than one of the enzymes in the system, it is possible to obtain improved fits to the observed data. However, doing so requires introducing additional free parameters that cannot be well identified. For this reason, a minimal model that satisfactorily explains the data within a reasonable error tolerance was developed.

An alternative hypothesis for the biophysical mechanism behind phosphate-dependent activation of the electron transport system is that phosphate modulates the redox coupling of cytochrome b and c, as proposed by Bose et al. [10]. It has been observed that binding to phosphate and other ions changes the apparent redox potential for cytochrome c [38]. These data on binding of cytochrome c and phosphate allow one to formalize this alternative hypothesis by appropriately modifying ΔG_o,C3_ and ΔG_o,C4_ to depend on phosphate concentration. However, a simple model (results not shown) using the linear relationship between the apparent cytochrome c redox potential and phosphate concentration observed by Gopal et al. [38] was unable to reproduce the phosphate-dependent behavior observed by Bose et al. [10]. Thus, the model detailed in this work represents the most parsimonious explanation that was found for the data illustrated in Figures 4 and 5.

In addition to reproducing the data used to parameterize the model, the mechanism of phosphate-dependent control of respiration matches observations in permeabilized cardiomyocytes indicating that phosphate represents the major feedback signal in low and medium work loads [7].

As noted above, the model remains unable to reproduce the observed data on membrane potential as phosphate concentration goes to zero. Note that as [Pi]_e_ → 0, the observed [NADH]/NAD_tot_ ratio, which is the driving force for electron transport and proton pumping, is approximately 0.6 for both levels of buffer ADP concentration that were studied (see Figure 4A). Yet the observed membrane potential, which is ultimately driven by the matrix redox state, is approximately 25 mV lower at [ADP]_e_ = 1.3 mM than at [ADP]_e_ = 0. Since there can be no ATP synthesis when [Pi]_e_ = 0, this behavior cannot be explained by a load on the F_1_F_0_ ATPase or the ANT.

It is expected that there always exists trace phosphate in the buffer and matrix and that nonviable mitochondria (e.g., mitochondria with compromised membranes) present in the experimental isolation act as ATPases, consuming ATP and generating phosphate [39]. Therefore, to obtain improved fits to the membrane potential data with ADP present at low phosphate concentration it may be appropriate to include an ATP-consuming reaction in the model of isolated mitochondria. However, introducing an ATP-consuming reaction into the model of isolated mitochondria has the effect of increasing the predicted oxygen consumption. With the current model it is not possible to reproduce both the difference in ΔΨ observed at [Pi]_e_ = 0 and the oxygen consumption curve by introducing an ATP-consuming reaction into the model (results not shown). Likely, the observed drop in membrane potential for [ADP]_e_ = 1.3 mM (and [Pi]_e_ = 0) compared to at [ADP]_e_ = 0 is due to residual phosphate in the bath and in the mitochondrial matrix, as proposed by Bose et al. [10].

Another mechanism that was considered as a possible explanation for the drop in ΔΨ observed when [ADP] is added to the buffer at [Pi]_e_ = 0 is that the activity of one or more the of the components of the electron transport system is enhanced when [ADP]_e_ = 0 compared to when [ADP]_e_ ≠ 0. Using the current model, one can obtain improved fits to the observed data by reducing the complex I or III activity, or by reducing the dehydrogenase activity, when [ADP]_e_ = 1.3 mM compared to the case when [ADP]_e_ = 0. Thus, inhibition of the activity of respiratory complexes by ADP represents a possible mechanism to explain the observations of Bose et al. [10] at [Pi]_e_ = 0. However, the present dataset does not provide enough information to determine which sites in the respiratory chain are modulated by the addition of ADP into the buffer. Further experiments are necessary to test competing hypotheses and to formulate a mechanistic model that explains the phenomenon in detail.

### Future Directions

As indicated above, the model remains imperfect and there remains room for improvement to model's fits to observed data. One potential avenue for improving the scope and predictive power of the model is to extend the model to include additional components of cardiac energetic metabolism. This task must be undertaken under the guidelines outlined in the Introduction. This operating philosophy behind model development requires that the use of purely data-driven empiricisms be avoided wherever possible. While nonphysical empirical relationships were not introduced in modeling the central components of the current model illustrated in Figure 1, the model is driven by an arbitrary four-parameter function (equation 1) used to represent overall dehydrogenase flux in the isolated system of Bose et al. [10]. This expression invokes a phenomenological dependence of the dehydrogenase flux on phosphate concentration, required to reproduce the observed data. In general, it is often difficult to avoid invoking such driving functions at the boundaries of a given model. Since it is planned to integrate the current model of oxidative phosphorylation with other components of cardiac metabolism [40], the data-driven dehydrogenase flux will be replaced with realistic models of the TCA cycle [12,13,40] and other reactions generating intracellular reducing potential [40]. While the current model was developed to analyze data from isolated mitochondria respiring on complex I substrates, such an integrated model would require a biophysical treatment of FAD(H_2_) redox handling at complex II of the respiratory chain. Thermodynamically balanced flux expressions for complex II flux could be developed in a manner analogous to that for complexes I and III in this study. These steps represent planned progress toward a major long-term goal of the construction of a complete energetic model of the cell [41].

Large-scale models of cellular energetics can be used for a variety of applications. For example, the current model may be linked with excitation–contraction models and calcium-dependent control of energy metabolism by extending the model to include sodium and calcium ion exchangers, as has been done in previous models [11,13,40]. Simulation and analysis of cardiac energetic requires integrating metabolic models into models of substrate transport at the cellular and tissue levels [42,43]. Thus, the current model provides a basis for integrating a detailed model of mitochondrial function into multiple-scale models of the heart.

While it was determined that the passive potassium channel flux is effectively zero in the model parameterization developed here, modeling the K^+^/H^+^ exchanger is required to buffer the matrix pH when both ΔΨ and [H^+^]_x_ are treated as state variables. Mitochondrial ATP-dependent potassium channel flux [17,18,37,44–47] will need to be incorporated to investigate the cardiac metabolic response to ischemia, hypoxia, and preconditioning. In addition, kinetic data must be obtained to effectively parameterize and validate the time-dependent behavior of the model. In sum, while a great number of extensions and improvements are possible, and many are planned, the current model represents the foundation for building larger and more complex systems and investigating complex physiological and pathophysiological interactions.

## Materials and Methods

The model was implemented, simulated, and analyzed using the MATLAB (The Mathworks, Natick, Massachusetts, United States) computing environment. All calculations were performed on a desktop PC. Computer codes are available from the author upon request. In addition, the model is available in the CellML exchange format at http://www.cellml.org.

## Abbreviations

AK: adenylate kinase
ANT: adenine nucleotide translocase
IM: intermembrane
MV_O2_: rate of oxygen consumption
PiHt: phosphate–hydrogen co-transporter
