# Correction: A Biophysical Model of the Mitochondrial Respiratory System and Oxidative Phosphorylation

**DOI:** 10.1371/journal.pcbi.0020008

**Published:** 2005-09-09

**Authors:** Daniel A Beard

In *PLoS Computational Biology,* volume 1, issue 4, DOI: 10.1371/journal.pcbi.0010036. Table 1 included incorrect values. The corrected table follows.

**Table 1:**
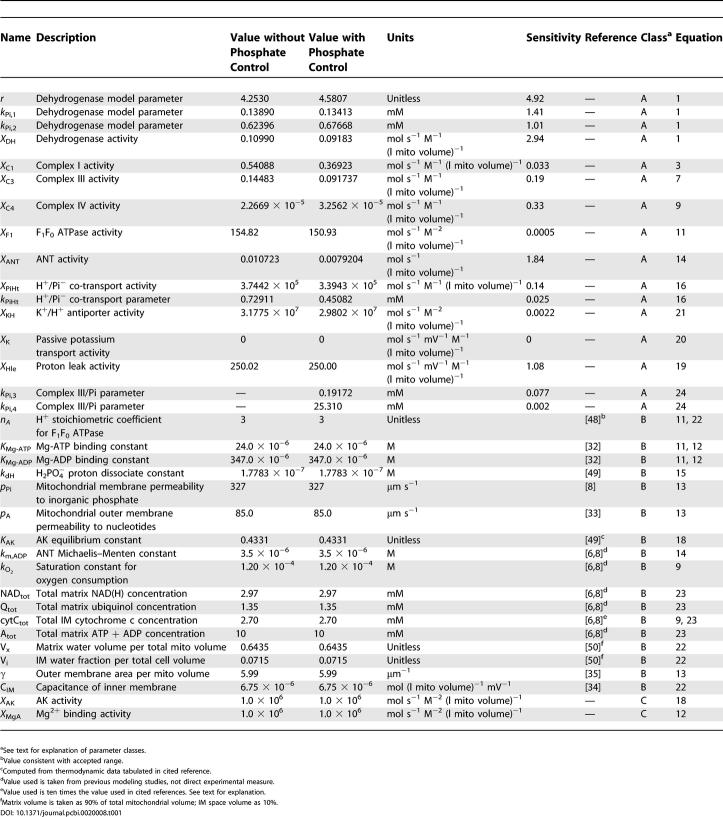
Mitochondrial Model Parameter Values

